# An Alternative Nutrient Rich Food Index (NRF-ai) Incorporating Prevalence of Inadequate and Excessive Nutrient Intake

**DOI:** 10.3390/foods10123156

**Published:** 2021-12-20

**Authors:** Bradley Ridoutt

**Affiliations:** 1 Commonwealth Scientific and Industrial Research Organisation (CSIRO) Agriculture and Food, Clayton, VIC 3169, Australia; brad.ridoutt@csiro.au; Tel.: +61-3-9545-2159; 2 Department of Agricultural Economics, University of the Free State, Bloemfontein 9300, South Africa

**Keywords:** affordability, Australian Health Survey, dairy foods, discretionary foods, ecoefficiency, ISO 14045, nutrient profiling, protein-rich foods, sustainable diet

## Abstract

Most nutrient profiling models give equal weight to nutrients irrespective of their ubiquity in the food system. There is also a degree of arbitrariness about which nutrients are included. In this study, an alternative Nutrient Rich Food index was developed (NRF-ai, where ai denotes adequate intake) incorporating prevalence of inadequate and excessive nutrient intake among Australian adults. Weighting factors for individual nutrients were based on a distance-to-target method using data from the Australian Health Survey describing the proportion of the population with usual intake less than the Estimated Average Requirement defined by the Nutrient Reference Values for Australia and New Zealand. All nutrients for which data were available were included, avoiding judgements about which nutrients to include, although some nutrients received little weight. Separate models were developed for females and males and for selected age groups, reflecting differences in nutrient requirements and usual intake. Application of the new nutrient profiling models is demonstrated for selected dairy products and alternatives, protein-rich foods, and discretionary foods. This approach emphasises the need to identify foods that are rich in those specific nutrients for which intake is below recommended levels and can be used to address specific nutrient gaps in subgroups such as older adults. In addition, the new nutrient profiling model is used to explore other sustainability aspects, including affordability (NRF-ai per AUD) and ecoefficiency (NRF-ai/environmental impact score).

## 1. Introduction

The food system is an important source of greenhouse gas emissions and other environmental impacts that at the global level are broadly viewed as unsustainable [1,2,3]. Poor diets are also linked to many diseases and health concerns [4]. The widespread adoption of healthy sustainable diets is therefore viewed as a way to address both of these inter-connected problems [5,6]. However, it can be difficult to draw general conclusions about what constitutes a healthy sustainable diet, as food production systems vary widely across different regions, as do specific environmental and public health nutrition challenges, as well as cultural dietary preferences. The evidence base is expanding rapidly [7,8,9,10]. However, in the opinion of some observers, many current recommendations about healthy sustainable diets are poorly substantiated and biased [11]. What is clear is that excessive consumption of discretionary (also called indulgence or noncore) foods can negatively impact diet quality and inflate dietary environmental burdens [12,13,14,15]. Efforts to moderate the intake of these foods, which are energy-dense and nutrient-poor, should be a priority. Suggestions to limit or exchange healthy food choices within a food group are more problematic as they have potential for unintended nutritional impacts [16,17]. Foods within the same food group can have very different nutritional profiles.

In this respect, nutrient profiling tools have the potential to support a transition to healthy sustainable diets by revealing nutritional trade-offs of proposed food substitutions. Nutrient profiling is the science-based approach to classifying and ranking foods according to nutritional composition [18]. Over the past two decades, a large number of such tools have been developed using a variety of different algorithms and including different numbers of included nutrients that are intended to be encouraged or limited [19]. The Nutrient Rich Food Index 9.3 [20,21] and the SAIN, LIM nutrient density score [22] are two examples. Nutrient density has been expressed relative to mass (e.g., 100 g), energy content (e.g., 100 kcal) or serving size [23] and also in relation to other sustainability measures such as affordability and environmental impact [24]. Some recent nutrient profiling tools have been adapted to include bioactive phytochemicals in addition to protein, fibre, vitamins, and minerals [25], and to align more closely with food-based dietary guidelines [26]. Much of the development of nutrient profiling tools has followed the emergence of nutrient density as a concept in national dietary guidelines [27] and the need for objective measures to regulate product health claims [28]. Other applications of nutrient profiling tools have included regulation of advertising, public health nutrition education, and new product development in the food industry.

Notwithstanding the progress that has been made in the science of nutrient profiling and the valuable ways in which they have been used, current nutrient profiling tools are not without limitations for some applications. Firstly, there is a degree of arbitrariness about the selection of nutrients that are included/excluded. For example, the NRF9.3 also has variants NRF6.3, 10.3, 11.3, and 15.3 that have varying numbers of included nutrients [23,29]. According to one review, the number of index nutrients in published models has ranged from 5 to 40 [24]. While included nutrients are typically justified on the basis of information contained in dietary guidelines, the choice of nutrient profiling model can clearly have a major influence on reported results. Secondly, nutrient profiling tools do not always reflect contemporary understanding of the role of specific nutrients in human nutrition, which is constantly evolving as new evidence is presented. For example, most models include saturated fats as a negative nutrient. However, today there is less consensus about this. One recent review concluded that saturated fatty acid-rich foods, such as whole-fat dairy, unprocessed meat, and dark chocolate, are not associated with an increased risk of cardiovascular disease, and that the totality of evidence does not support limiting their intake [30]. Thirdly, existing nutrient profiling models typically give equal weight to all included nutrients, regardless of the degree to which intake is inadequate or excessive. Fourthly, nutrient profiling models tend to be rather generic and there is further potential for adaptation to reflect the importance of specific nutrients to specific population groups, as well as the different nutrient requirements of women and men across life stages.

In this research, a new variation of the Nutrient Rich Food Index (NRF-ai, where ai denotes adequate intake) was developed for Australian adults using weighting factors for individual nutrients based on prevalence of inadequate and excessive intake in the population. This approach emphasises the need to identify foods that are rich in those specific nutrients for which intake is below recommended levels. Some nutrients, although very important, are sufficiently prevalent in the food system that intake is already adequate or close to adequate. The potential of this approach has been mentioned often in the literature [19,20,23,29,31]. In this study, the approach is operationalised for Australian adults and for selected subgroups. Examples are also used to demonstrate how the new index can be applied in relation to the affordability and environmental impact aspects of sustainability.

## 2. Materials and Methods

### 2.1. Development of Weighting Factors

The National Nutrition and Physical Activity Survey component [32] of the Australian Health Survey [33] is a large and nationally representative assessment undertaken by the Australian Bureau of Statistics (ABS). Dietary intake data were collected for more than 9000 adults using a 24 h recall method and a complex sampling process to enable the estimation of intake of foods and nutrients for the Australian population as well as demographic subgroups through the application of population weighting factors. From these data, the ABS has published tables of usual nutrient intakes [34] as well as the proportion of the population within each subgroup with usual intake less than the Estimated Average Requirement (EAR) described in the Nutrient Reference Values for Australia and New Zealand [35] (see Supplementary Table S1 for details). The population subgroups were females and males in age groups 19–30, 31–50, 51–70 and above 70 years. These data cover eight vitamins (B1, B2, B3, B6, B12, Folate, A, and C) and eight minerals (Ca, P, Zn, Fe, Mg, I, Se, and Mo), along with protein and free sugars. Regarding protein, inadequate intake is reported relative to the lower limit of acceptable macronutrient distribution range (i.e., 15% of energy). For free sugars, excessive intake is reported when the proportion of energy intake reaches or exceeds 10%. The data for prevalence of inadequate or excessive nutrient intake that were used in this study were obtained directly from these sources.

To develop the new Nutrient Rich Food Index (NRF-ai), all of the above-mentioned nutrients were included. No decision was taken to arbitrarily exclude any nutrients, fulfilling a key criterion that the index is science-based [19,20] and as free as possible from potential bias introduced through model design. Regarding sodium, Australia’s National Health and Medical Research Council undertook a review of the evidence and in 2017 amended the nutrient reference values (NRVs) such that the upper level of intake is now not determined [35]. Similarly, the NRVs do not include a specific upper level of intake for saturated fats. Regarding excessive intake of typically beneficial vitamins and minerals, this is almost non-existent according to data from the National Nutrition and Physical Activity Survey [34]. Intakes above the upper levels were reported only for phosphorus at a level of 0.1% for males, which was deemed to be trivial.

As the prevalence of inadequate or excessive nutrient intake is uneven across nutrients, a weighting model was developed using a distance-to-target method, where the relative distance to target determines the weight. This is a common science-based approach used widely in the environmental sciences [36]. In this case, nutrients were weighted in direct proportion to the extent of inadequate or excessive intake, e.g., a nutrient with a prevalence of inadequate intake of 20% was assigned a weighting of 0.20. Unique weighting models were developed for each age and gender subgroup. Weighting models were also developed that combined age groups using recent ABS population estimates [37]. Finally, the weighting factors were scaled in order to sum to 1.00.

### 2.2. Application to Selected Foods

Data describing the nutrient content of foods (per 100 g) and beverages (per 100 mL) in the Australian food system were obtained from the Australian Food Composition Database [38]. Some nutrients are only required in μg levels, whereas others are required in larger quantities (mg or g). As such, the data describing the nutrient content of foods were transformed to express fraction of EAR per 100 g or 100 mL [35]. Separate calculations were performed for the various age and gender subgroups having different EARs. As the purpose of this research was to identify foods that are rich in those specific nutrients for which intake tends to be inadequate or excessive, the nutrient contents of foods were subsequently expressed per serving size as described in the Australian Dietary Guidelines [39] (see Supplementary Table S2 for details) For example, a standard serving is 75 g for vegetables, 150 g for fresh fruit, 30 g for dried fruit and 125 mL for fruit juice, etc. Of course, the actual quantity an individual consumes as a serving may deviate from these values. However, these values are indicative of the amounts customarily consumed and form a basis for realistic comparison between foods within the same food group which have substantially different moisture content. For example, a standard serving of milk, soy, nut, rice, or other cereal beverage is 250 mL, whereas a standard serving of hard cheese, such as cheddar, is 40 g and for a soft cheese such as ricotta is 120 g. To calculate the NRF-ai for each food, the 18 included nutrients (fraction of EAR per serve) were multiplied by the respective weighting factors described above. The final values were obtained by summing the weighted scores, with a negative value attributed to sugar.

### 2.3. Additional Sustainability Indicators

To investigate the affordability of foods that address nutrient gaps, the ratio of NRF-ai score to retail price was explored. Data describing the retail price of foods were obtained from major Australian supermarket websites [40,41]. Where a variety of products were available, the lowest prices were selected, excluding temporary price reductions. Additionally, the ecoefficiency of foods that address nutrient gaps was explored by calculating the ratio of NRF-ai score to environmental impact score following ISO 14045 [42]. In previous research, lifecycle assessment was used to quantify the climate footprints (carbon dioxide equivalents), water-scarcity footprints (litre equivalents), and cropland-scarcity footprints (m^2^ year equivalents) of a large number of processed and unprocessed foods in the Australian food system [43,44,45]. These data have been used to develop an environmental impact score for each food [15]. The specific weighting factors were 0.585 for climate footprint, 0.294 for water-scarcity footprint, and 0.121 for cropland-scarcity footprint, based on planetary boundary targets. Detailed information about the data, equations, and assumptions underpinning the environmental modelling is available in the associated references.

## 3. Results

### 3.1. Model Coefficients

The prevalence of inadequate and excessive nutrient intake is variable across nutrients and across population subgroups in Australia. As such, weighting models were developed using a distance-to-target approach where the weight given to individual nutrients reflected the extent of inadequacy or excess. The resulting weighting factors, scaled such that they sum to 1.00, are presented in Table 1 (and Supplementary Table S3). When considering the entire Australian adult population, calcium received the highest weight (0.22), followed by free sugars (where intake is in excess), magnesium, vitamin B6 and zinc. Together, these five nutrients accounted for more than 75% of the total weighting. However, it is important to underscore that these five nutrients were not chosen a priori but emerged from the impartial weighting process. Additionally, the other 13 nutrients were not excluded. However, they received lesser weights, reflecting prevalence of inadequate intake at much lower levels across the adult population.

Differences in weighting factors were also evident across population subgroups (Table 1). For example, the weighting factor for free sugars was 0.20 for the 19 to 30-year age subgroup, but only 0.11 for adults above 70. Weighting factors also differed between females and males. In the case of zinc, where the EAR is much higher for males than females (12.5 mg per day compared to 6.5 mg per day) and the prevalence of inadequate intake is also much higher in males (45.7% compared to 10.5% for females), the weighting factor was 0.17 for males (the third highest ranked nutrient), compared to only 0.03 for females (the ninth highest ranked nutrient).

### 3.2. Nutrient Profiling Scores

**Figure 1:**
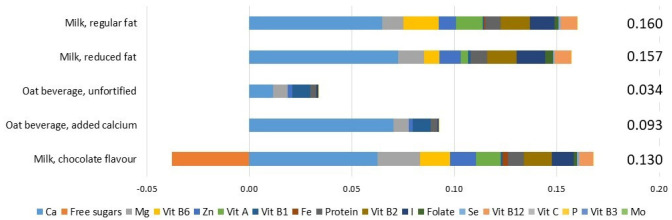
Examples of NRF-ai scores for Australian adults (19 years and above). Scores are per serving, 250 mL in all cases.

The weighting factors (Table 1) can be applied to any food or beverage product for which the nutrient composition is known. Figure 1 presents the Nutrient Rich Food Index scores for a selection of dairy foods and non-dairy alternatives. These examples were chosen as they include variations with fortification and added sugar. Among these products, regular-fat milk received the highest nutrient profile score (0.160 per serving, 250 mL), with the largest contributions from calcium and vitamins A, B2 and B6. Reduced-fat milk received a marginally lower score. Unfortified oat beverage, which might be consumed as a substitute for cow’s milk but does not qualify as a dairy-alternative under the Australian Dietary Guidelines as it does not contain at least 100 mg Ca per 100 mL [39], received the lowest score. Oat beverage with added calcium received a higher score than unfortified oat beverage, however this was still much lower than cow’s milk. For fortified oat beverage, the added calcium contributed 76% of the total nutrient profile score. Chocolate-flavoured milk provided similar nutrients to regular milk; however, the added sugar reduced the total score to 0.130, which was around 20% lower than regular milk, but still substantially higher than calcium-fortified oat beverage.

For discretionary or indulgence foods, nutrient profile scores were, as expected, very low or even negative. For example, a chocolate chip cookie scored 0.004 per 35 g serve. A serving of sugar-sweetened cola flavoured drink (375 mL) scored −0.135. Commercial muesli bars scored between 0.018 and 0.021, depending on variety. Such low scores are consistent with their characterisation in the Australian Dietary Guidelines as discretionary foods. See Supplementary Table S4 for an expanded list of foods.

**Figure 2:**
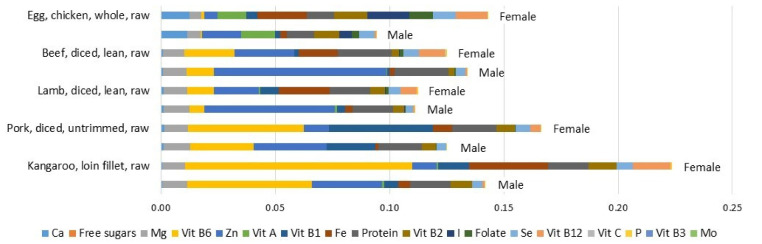
Comparison of NRF-ai scores for a selection of protein-rich foods for Australian adult (19 years and above) females and males. Scores are per serving, 95 g raw for red meat and 2 large eggs without shell.

Nutrient profile scores were also found to vary between females and males for the same foods, as shown in Figure 2 for a selection of protein-rich foods. For example, the nutrient profile score for eggs was higher for females (0.143) than males (0.094). In part, this is explained by the higher weighting factors for iron and vitamin B12 in the model applied to females (Table 1). In the case of beef, the nutrient profile scores were marginally lower for females (0.125) than males (0.134). In part, this is explained by the higher weighting factor applied to zinc in the model applied to males (Table 1).

### 3.3. Affordability and Ecoefficiency

**Figure 3:**
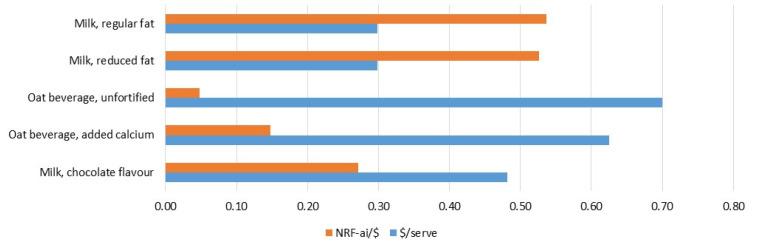
Nutrient-Rich Food Index (NRF-ai) per Australian $ (AUD) and retail price per serving for a selection of Australian dairy foods and non-diary alternatives. Nutrient density calculation incorporates prevalence of inadequate and excessive nutrient intake across Australian adults (19 years and above). Serving size is 250 mL in all cases.

Figure 3 presents the retail prices (per serving) for the same selection of dairy and non-dairy foods shown in Figure 1. Considering only the lowest priced products advertised by major grocery retailers in Melbourne, Australia (excluding products on temporary price reduction), milk (regular or reduced fat) was found to be the most affordable (AUD 0.30 per serving). The least affordable of this selection was unfortified oat beverage (AUD 0.70 per serving). As such, among these five alternatives, cow’s milk was by far the most affordable means of addressing nutrient gaps in the Australian adult population (Figure 3).

**Figure 4:**
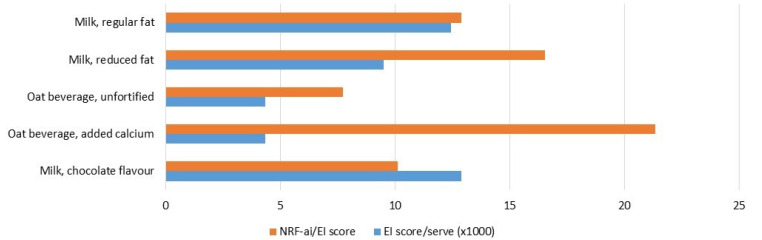
Nutrient-Rich Food Index (NRFai) per Environmental Impact score (EI score; higher score describes higher impact) and EI score per serving for a selection of Australian dairy foods and non-diary alternatives. Nutrient density calculation incorporates prevalence of inadequate and excessive nutrient intake across Australian adults (19 years and above). Serving size is 250 mL.

Environmental impact is an additional sustainability consideration. In Figure 4, a multidimensional environmental impact score (EI score) is reported for these same five products. The EI scores ranged from 0.013 for chocolate flavoured milk to 0.004 for oat beverage (per serving, 250 mL), obtained from a previous study [15]. When expressed as ecoefficiency (NRF-ai score/EI score), oat beverage with added calcium attained the highest value of 21.3. In other words, among these products, calcium fortified oat beverage was the most environmentally efficient means of supplying nutrients that address nutrient gaps in Australian adults. The next most eco-efficient product, among this selection of five, was reduced-fat milk, scoring 16.5. While reduced-fat milk has almost the same nutrient profile score as milk with regular fat, the EI score is lower than regular milk because part of the environmental burden associated with production is allocated to the products that are made from the removed fat. Among this selection of products, unfortified oat milk obtained the lowest ecoefficiency score (7.7).

## 4. Discussion

In Australia, as in many other parts of the world, dietary guidance differs for females and males and across different age groups, reflecting the available scientific evidence with regard to the role of nutrients in promoting health and wellbeing and reducing the risk of deficiencies and chronic disease. It seems beneficial that nutrient profiling tools should also have this potential for differentiation. As such, a new family of Nutrient-Rich Food indexes (NRF-ai) was developed, incorporating age- and gender-specific data for inadequate and excessive nutrient intake. Although there were high degrees of correlation between the individual nutrient profiling models (R = 0.78 and above), there were also important differences, such as differences in weighting applied to free sugars (0.11 to 0.20), zinc (0.03 to 0.17) and iron (0.01 to 0.07) (Table 1). Such differentiation could support the development of new food products that are designed to cater to the needs of specific population subgroups. One example could be foods that address the specific nutrient needs of older adults [46,47] or women. Tailored nutrient profiling models could also support the regulation of marketing and health claims that are directed toward specific population subgroups, as well as public health nutrition education more generally. While there is a variety of age-group- and gender-defined nutrient profiling models presented in this article (Table 1), the same approach could be used to develop a unique model for any subgroup of interest. This could include people living in indigenous communities [48,49].

Another important consideration is impartiality in nutrient profiling model design. As noted already, the number of included nutrients varies greatly across published models, from as few as 5 to as many as 40 [24]. To avoid potential conflicts of interest, it is desirable that nutrient profiling models are evidence-based with as few researcher-based choices in the model design, especially in regard to which nutrients to include or exclude. Arsenault et al. demonstrated a statistical approach to selecting and weighting nutrients that used coefficients from a regression model associating nutrients with Healthy Eating Index scores [50]. An eight-nutrient model that explained 65% of the variance was recommended. Here, it is important to note that the Healthy Eating Index is itself a construct designed to assess diet quality, and does not provide certainty of adequate intake of any particular nutrient. In the present study, the NRF-ai used dietary intake survey data reporting level of prevalence of inadequate and excessive nutrient intake. As such, it is more directly related to published Nutrient Reference Values. This is of critical importance in the context of sustainable diets. Recommendations to reduce the environmental impacts of diets typically involve substitutions of foods within a food group. In Australia, as in many countries, officially dietary guidelines are based on recommended servings within food groups [39]. The modelling that supported the design of Australia’s dietary guidelines was based on customary food choices within each food group [51]. As such, the guidelines emphasize variety of food choice within a food group. Although it is nominally feasible to comply with the guidelines by consuming all of the recommended servings of vegetables as only carrots or all of the recommended servings of protein foods as only eggs, this is not the way the guidelines are intended to be applied and is likely to result in nutrient gaps. Therefore, recommendations to narrow food choice by excluding foods that are deemed to have a higher environmental footprint have the potential to compromise nutrient intake. For example, Figure 2 illustrates how for females, eggs have a higher nutrient density than diced lamb or beef, but this is not the case for males. It is to be noted that this comparison is based on nutrient composition before cooking and this assessment could change depending on method of food preparation. Nevertheless, this example highlights the potential nutritional trade-offs that are possible when sustainability considerations are brought into dietary recommendations. Nutrient profiling tools that are age-group- and gender-specific and based on prevalence of inadequate and excessive nutrient intake could help address these risks.

This study has shown that nutrient density is not necessarily related to retail price (Figure 3), confirming previous evidence [24,31]. Comparing various dairy and non-dairy alternatives, on the day of the survey the most expensive option was unfortified oat beverage, which was also the least nutrient dense. Here, it is important to note the observation that unlike fluid milk, non-dairy alternatives are frequently subject to temporary price reductions in supermarkets in Australia. This suggests that the margins between production cost and ordinary price may be large for these types of products. In any case, the data presented in Figure 3 demonstrate the potential of NRF-ai to be used to address policy related questions around affordable nutrition.

In terms of ecoefficiency (NRF-ai score/EI score), oat beverage with added calcium scored highest among the dairy products and alternatives considered (Figure 4). Although having a lower nutrient density compared to cow’s milk, oat beverage has a much smaller environmental impact per serving. As such, fortified oat beverage is a highly environmentally efficient way of obtaining the limited range of nutrients (mainly Ca, Figure 1) it contains. This raises interesting questions about the role of dairy alternatives in a sustainable diet. In Australia, total intake of dairy foods depends substantially on the number of eating occasions where dairy foods are included [52]. What this means is that if dairy-eating occasions include less nutrient-dense dairy alternatives, there is a need to make up for the “lost” nutrients elsewhere in the diet. Unfortunately, the evidence in relation to dairy avoiders and low-dairy-food consumers in Australia suggests that this is not ordinarily the case [17]. This would appear to be an area where further public health nutrition research and potentially intervention is required. As noted by Green et al., the combined assessment of the nutritional and environmental aspects of foods is not straightforward [53,54]. Composite indices are attractive in the sense that they combine two or more sustainability aspects (in this case, nutrient density and environmental impact) into a single score; however, in doing so transparency is lost, and there is potential for unintended negative consequences. When one food is chosen, typically it replaces some alternative in the diet and the full implications can only be understood in the context of complete dietary patterns. For these reasons, ecoefficiency may be more appropriately assessed at the level of complete diets rather than at the level of individual foods. There are also questions about which aspects of sustainability (human health, affordability, environmental sustainability, etc.) should be given the most emphasis in delivering a sustainable diet and how the inevitable trade-offs should be managed.

Nutrient profile scores have been expressed relative to mass, volume, energy content, average portion size and serving size. The main issue is that the formulation should be relevant in a specific context. In this study, results are expressed per serving size as described in the Australian Dietary Guidelines. The intention here was to align with amounts that are indicative of what might usually be consumed. This approach also aligns with mandatory labelling requirements in Australia for packaged foods which need to include nutrient composition per serving size. That said, the Australian Dietary Guidelines do not provide a comprehensive list of serving sizes for all foods. In addition, the serving sizes described in the guidelines are not always the same as those adopted by food manufacturers. In this study, examples were chosen to avoid these complications. For example, dairy foods and alternatives were all compared on a 250 mL basis. However, the practical application of nutrient profiling tools for product comparisons depends on a consistent definition of a serving size. The Australian Dietary Guidelines describe a serving of yoghurt as 200 g. However, many individually packaged yoghurts in Australia are more in the size range of 110 to 150 g. If packaged foods are assessed relative to their declared serving size, this means that standard servings only need to be defined for unprocessed foods and items sold in bulk quantities, limiting the problem somewhat, although it is acknowledged that there is no perfect solution.

In this study, all nutrients were included for which an EAR is published in Australia by the National Health and Medical Research Council [35]. As such, the NRF-ai nutrient profiling tool does not include all nutrients that are beneficial or potentially deleterious for health. For example, the model does not include dietary fibre. According to the National Health and Medical Research Council in Australia, adequate dietary fibre is essential for proper functioning of the gut and has been related to risk reduction for several chronic diseases; however, assessment of dietary fibre needs is complex, and the endpoints are ill-defined, meaning that it is not considered possible to currently establish an EAR. In future, if an EAR is determined for dietary fibre or any other additional nutrient in Australia, the NRF-ai can be amended accordingly, and thereby retain consistency with the best available evidence, as determined by responsible authorities. Similarly, the NRF-ai does not include consideration of sodium since there is no defined upper limit for adults in Australia. This could be viewed as a limitation. However, the NRF-ai was clearly able to identify nutrient-poor discretionary foods, many of which contain higher levels of added sodium. As such, the exclusion of sodium may not limit the usefulness of the index. While it is broadly acknowledged that most Australians would benefit from reduced sodium intake [35], the absence of a defined upper level of intake in Australia precludes the inclusion of sodium in the NRF-ai. Should an upper level of intake be defined in the future, the NRF-ai could be revised. In a similar way, the NRF-ai does not include consideration of protein quality [55,56,57], bioavailability [58,59], or nutrient–nutrient interactions [60,61], which are known to differ between foods. However, these aspects could be included in future when sufficient evidence exists for their role in nutrition to be acceptably quantified. Finally, there is the potential that preference for foods with higher NRF-ai scores could lead to lower intake of those nutrients for which intake is presently adequate. This is unlikely since foods that score highly on the NRF-ai are generally nutrient dense and also include other nutrients such as those that are more ubiquitous in the food system. That said, this is something that could be monitored.

## 5. Conclusions

The alignment between nutrient profiling tools and dietary guidelines can be improved by developing customised models for population subgroups defined by age-group, gender, or any other basis for which information about nutrient intake is available. A nutrient profiling model that weights nutrients according to prevalence of inadequate and excessive nutrient intake in the population of interest also avoids subjective decisions about which and how many nutrients to include, as these decisions have the potential to undermine confidence in nutrient profiling tools. Such weighting also has the benefit of emphasising those nutrients which are less abundant in the general food system.

## Figures and Tables

**Table 1:** Nutrient weighting factors applicable for Australian adults and adult subgroups. Columns sum to 1.00.

Nutrient	Population Subgroup						
	19–30 y	31–50 y	51–70 y	70+ y	Females 19+ y	Males 19+ y	Adults 19+ y
Calcium	0.20	0.20	0.26	0.24	0.24	0.20	0.22
Free sugars	0.20	0.17	0.12	0.11	0.14	0.18	0.15
Magnesium	0.12	0.13	0.13	0.14	0.11	0.15	0.13
Vitamin B6	0.07	0.08	0.16	0.17	0.14	0.08	0.12
Zinc	0.09	0.09	0.10	0.09	0.03	0.17	0.09
Vitamin A	0.07	0.06	0.05	0.04	0.05	0.07	0.05
Vitamin B1	0.04	0.05	0.05	0.04	0.06	0.03	0.05
Iron	0.07	0.07	0.01	0.01	0.07	0.01	0.04
Protein	0.05	0.04	0.04	0.03	0.03	0.05	0.04
Vitamin B2	0.02	0.02	0.03	0.05	0.03	0.03	0.03
Iodine	0.02	0.02	0.02	0.02	0.03	0.01	0.02
Folate	0.02	0.02	0.02	0.01	0.03	0.01	0.02
Selenium	0.01	0.01	0.02	0.03	0.02	0.01	0.02
Vitamin B12	0.01	0.01	0.01	0.01	0.02	0.00	0.01
Vitamin C	0.01	0.01	0.01	0.01	0.01	0.01	0.01
Phosphorus	0.00	0.00	0.00	0.00	0.00	0.00	0.00
Vitamin B3	0.00	0.00	0.00	0.00	0.00	0.00	0.00
Molybdenum	0.00	0.00	0.00	0.00	0.00	0.00	0.00

y: years old.

## Data Availability

The dietary intake data and tables of usual nutrient intakes are available from the Australian Bureau of Statistics (http://www.abs.gov.au). Nutrient reference values are available from the National Health and Medical Research Council (https://www.nrv.gov.au/).

## Supplementary Materials

The following are available online at https://www.mdpi.com/article/10.3390/foods10123156/s1, Table S1: Daily Estimated Average Requirement (EAR) for nutrients according to age and gender subgroups; Table S2: Examples of standard serves of food described in the Australian Dietary Guidelines; Table S3: Nutrient weighting factors applicable for Australian adults and adult subgroups; Table S4: Examples of NRF-ai scores for common foods in the Australian food system.
