# Supplementary material for: An Alternative Nutrient Rich Food Index (NRF-ai) Incorporating Prevalence of Inadequate and Excessive Nutrient Intake
Source: Foods. 2021 Dec 20;10(12):3156. doi: 10.3390/foods10123156 (PMC8701859; doi:10.3390/foods10123156)
Supplement: Supplementary file 1 [file foods-10-03156-s001.zip › foods-1471495-supplementary.pdf]

Supplementary Materials

An alternative Nutrient Rich Food index (NRF-ai) incorporating prevalence of inadequate and excessive nutrient intake

Bradley Ridoutt

Supplementary Table S1. Daily Estimated Average Requirement (EAR) for nutrients according to age and gender subgroups.

Nutrient	Unit	EAR							
		Females				Males			
		19-30	31-50	50-70	70+	19-30	31-50	50-70	70+
Protein	g	37	37	37	46	52	52	52	65
Vitamin B1	mg	0.9	0.9	0.9	0.9	1	1	1	1
Vitamin B2	mg	0.9	0.9	0.9	1.1	1.1	1.1	1.1	1.3
Vitamin B3[1]	mg	11	11	11	11	12	12	12	12
Vitamin B6	mg	1.1	1.1	1.3	1.3	1.1	1.1	1.4	1.4
Vitamin B12	µg	2	2	2	2	2	2	2	2
Folate[2]	µg	320	320	320	320	320	320	320	320
Vitamin A[3]	µg	500	500	500	500	625	625	625	625
Vitamin C	mg	30	30	30	30	30	30	30	30
Calcium	mg	840	840	1,100	1,100	840	840	840	1,100
Phosphorus	mg	580	580	580	580	580	580	580	580
Zinc	mg	6.5	6.5	6.5	6.5	12	12	12	12
Iron	mg	8	8	5	5	6	6	6	6
Magnesium	mg	255	265	265	265	330	350	350	350
Iodine	µg	100	100	100	100	100	100	100	100
Selenium	µg	50	50	50	50	60	60	60	60
Molybdenum	µg	34	34	34	34	34	34	34	34

[1] Niacin equivalents; [2] Dietary folate equivalents; [3] Retinol equivalents. All nutrients for which an EAR is quantified in Australia by the National Health and Medical Research Council (NHMRC) are included. Data sourced from https://www.nrv.gov.au/

Supplementary Table S2. Examples of standard serves of food described in the Australian Dietary Guidelines

**Food description**	**Unit**	**Standard serve**
Vegetables	g	75
Fruit (fresh)	g	150
Fruit (juice)	ml	125
Fruit (dried)	g	30
Milk	ml	250
Soy, rice, nut, or cereal beverage	ml	250
Cheese (firm)	g	40
Cheese (soft, such as Ricotta)	g	120
Ice-cream	g	75
Eggs	g	120
Legumes/beans (soaked, cooked)	g	150
Tofu	g	170
Nuts	g	30
Meat (red, cooked)	g	65
Meat (poultry, cooked)	g	80
Fish (cooked)	g	100
Bread	g	40
Commercial breakfast cereal	g	30
Beverage (sugar or artificial sweetener)	ml	375
Biscuit (sweet)	g	35
Salty cracker or crisps	g	30
Chocolate	g	25

Data sourced from https://www.eatforhealth.gov.au/guidelines

Supplementary Table S3. Nutrient weighting factors applicable for Australian adults and adult subgroups.

	Females 19-30	Males 19-30	All 19-30	Females 31-50	Males 31-50	All 31-50	Females 51-70	Males 51-70	All 51-70	Females 70+	Males 70+	All 70+	Males 19+	Females 19+	All 19+
Calcium	0.20	0.19	0.20	0.21	0.19	0.20	0.30	0.21	0.26	0.25	0.22	0.24	0.20	0.24	0.22
Free sugar	0.17	0.25	0.20	0.14	0.22	0.17	0.12	0.13	0.12	0.11	0.12	0.11	0.18	0.14	0.15
Magnesium	0.10	0.15	0.12	0.10	0.16	0.13	0.10	0.16	0.13	0.13	0.16	0.14	0.15	0.11	0.13
Vitamin B6	0.10	0.02	0.07	0.12	0.04	0.08	0.19	0.13	0.16	0.19	0.14	0.17	0.08	0.14	0.12
Zinc	0.04	0.16	0.09	0.03	0.17	0.09	0.03	0.17	0.10	0.03	0.16	0.09	0.17	0.03	0.09
Vitamin A	0.06	0.09	0.07	0.05	0.08	0.06	0.04	0.06	0.05	0.04	0.03	0.04	0.07	0.05	0.05
Thiamin(B1)	0.05	0.03	0.04	0.06	0.03	0.05	0.06	0.03	0.05	0.05	0.02	0.04	0.03	0.06	0.05
Iron	0.10	0.01	0.07	0.12	0.01	0.07	0.02	0.01	0.01	0.02	0.01	0.01	0.01	0.07	0.04
Protein	0.05	0.05	0.05	0.04	0.05	0.04	0.03	0.04	0.04	0.02	0.04	0.03	0.05	0.03	0.04
Riboflavin(B2)	0.02	0.01	0.02	0.02	0.02	0.02	0.03	0.03	0.03	0.05	0.05	0.05	0.03	0.03	0.03
Iodine	0.03	0.01	0.02	0.03	0.01	0.02	0.03	0.01	0.02	0.02	0.01	0.02	0.01	0.03	0.02
Folate	0.03	0.01	0.02	0.03	0.01	0.02	0.02	0.01	0.02	0.02	0.00	0.01	0.01	0.03	0.02
Selenium	0.02	0.01	0.01	0.02	0.01	0.01	0.02	0.01	0.02	0.03	0.03	0.03	0.01	0.02	0.02
Vitamin B12	0.02	0.00	0.01	0.02	0.00	0.01	0.02	0.00	0.01	0.02	0.00	0.01	0.00	0.02	0.01
Vitamin C	0.01	0.01	0.01	0.01	0.01	0.01	0.01	0.00	0.01	0.01	0.00	0.01	0.01	0.01	0.01
Phosphorus	0.00	0.00	0.00	0.00	0.00	0.00	0.00	0.00	0.00	0.00	0.00	0.00	0.00	0.00	0.00
Niacin(B3)	0.00	0.00	0.00	0.00	0.00	0.00	0.00	0.00	0.00	0.00	0.00	0.00	0.00	0.00	0.00
Molybdenum	0.00	0.00	0.00	0.00	0.00	0.00	0.00	0.00	0.00	0.00	0.00	0.00	0.00	0.00	0.00

Columns sum to 1.00.

Supplementary Table S4. Examples of NRF-ai scores for common foods in the Australian food system.

DRIED FRUITS	SERVING		NRF-ai score
Apricot, dried	30	g	0.037
Date, dried	30	g	0.024
Mixed dried fruit	30	g	0.012
Prune (dried plum)	30	g	0.023
Sultana	30	g	0.022
FRESH FRUITS	SERVING		NRF-ai score
Apple, red skin, unpeeled, raw	150	g	0.020
Apricot, raw	150	g	0.050
Banana, cavendish, peeled, raw	150	g	0.067
Blueberry, raw	150	g	0.016
Grape, red globe, raw	150	g	0.024
Grapefruit, peeled, raw	150	g	0.037
Kiwifruit, gold, peeled, raw	150	g	0.082
Mandarin, peeled, raw	150	g	0.058
Mango, peeled, raw	150	g	0.058
Melon, watermelon, peeled, raw	150	g	0.022
NUTS	SERVING		NRF-ai score
Nut, almond, with skin, roasted, unsalted	30	g	0.083
Nut, brazil, raw or blanched, unsalted	30	g	0.180
Nut, cashew, raw, unsalted	30	g	0.090
Nut, hazelnut, raw, unsalted	30	g	0.069
Nut, macadamia, raw, unsalted	30	g	0.039
Nut, peanut, with skin, raw, unsalted	30	g	0.081
Nut, pecan, raw, unsalted	30	g	0.053
Nut, pine, raw, unsalted	30	g	0.069
Nut, pistachio, raw, unsalted	30	g	0.095
Nut, walnut, raw, unsalted	30	g	0.062
VEGETABLES	SERVING		NRF-ai score
Asparagus, green, boiled, drained	75	g	0.039
Avocado, raw	75	g	0.033
Bean, green, fresh, boiled, drained	75	g	0.030
Beetroot, canned in brine, drained	75	g	0.020
Bok choy, steamed	75	g	0.078
Broccoli, fresh, boiled, drained	75	g	0.045
Carrot, mature, peeled, fresh, boiled, drained	75	g	0.138
Celery, fresh, raw	75	g	0.021
Cucumber, common, unpeeled, raw	75	g	0.024
Kale, raw	75	g	0.188
MUSHROOMS	SERVING		NRF-ai score
Mushroom, common, fresh, raw	75	g	0.016
Mushroom, common, fresh, fried, no added fat	75	g	0.027
GRAIN (CEREAL) FOODS	SERVING		NRF-ai score
Bread, from white flour	40	g	0.051
Bread, from white flour, added iron	40	g	0.070
Bread, from wholemeal flour	40	g	0.061
Bread, mixed grain	40	g	0.063
Bread, from rye flour, sour dough	40	g	0.034
Breakfast cereal, flakes of corn, unfortified	30	g	0.007
Breakfast cereal, flakes of corn, added vitamins B1, B2, B3, C & folate, Fe & Zn	30	g	0.078
Breakfast cereal, mixed grain (wheat, oat & corn), extruded, added vitamins B1, B2, B3, B6, C & folate, Ca & Fe	30	g	0.138
Muesli, toasted, added dried fruit & nuts, unfortified	30	g	0.051
Muesli, untoasted or natural style, added dried fruit, unfortified	30	g	0.038

Table continued on next page

Supplementary Table S4 continued

DAIRY FOODS AND ALTERNATIVES	SERVING		NRF-ai score
Milk, cow, fluid, regular fat (3.5% fat)	250	ml	0.160
Milk, cow, fluid, reduced fat (1% fat)	250	ml	0.157
Oat beverage, fluid, unfortified	250	ml	0.034
Oat beverage, fluid, added calcium	250	ml	0.093
Rice beverage, fluid, added calcium	250	ml	0.067
Soy beverage, regular fat (3% fat), unfortified	250	ml	0.056
Soy beverage, regular fat (3% fat), added Ca	250	ml	0.120
Beverage, chocolate flavour, from drinking chocolate, with regular fat cows milk	250	ml	0.130
Yoghurt, natural, regular fat (3% fat)	200	g	0.168
Yoghurt, apricot pieces or flavoured, regular fat (3% fat)	200	g	0.096
Yoghurt, strawberry pieces or flavoured, regular fat (3% fat)	200	g	0.116
Yoghurt, vanilla flavoured, (2% fat)	200	g	0.121
Yoghurt, vanilla flavoured, low fat (less than 0.5% fat)	200	g	0.125
Yoghurt, soy based, berry flavoured, regular fat (3% fat)	200	g	0.050
Yoghurt, soy based, vanilla flavoured, reduced fat (1% fat)	200	g	0.048
Cheese, camembert	40	g	0.105
Cheese, cheddar, natural, regular fat	40	g	0.124
Cheese, cheddar, processed, regular fat	40	g	0.103
Cheese, goat, firm	40	g	0.069
Cheese, soy	40	g	0.033
SPREADS	SERVING		NRF-ai score
Peanut butter, smooth & crunchy, added sugar & salt	10	g	0.018
Peanut butter, smooth & crunchy, no added sugar or salt	10	g	0.019
Butter, plain, salted	10	g	0.009
Margarine spread, polyunsaturated (70% fat)	10	g	0.007
Oil, olive	8	g	0.000
PROTEIN-RICH FOODS	SERVING		NRF-ai score
Tofu (soy bean curd), firm, as purchased	170	g	0.306
Egg, chicken, whole, hard-boiled	120	g	0.116
Egg, chicken, whole, poached	120	g	0.136
Egg, chicken, whole, fried, no fat added	120	g	0.151
Chickpea, canned, drained	150	g	0.105
Salmon, Atlantic, fillet, grilled, no added fat	100	g	0.134
Salmon, Atlantic, fillet, steamed, no added fat	100	g	0.133
Barramundi, aquacultured, fillet, grilled, no added fat	100	g	0.136
Barramundi, aquacultured, fillet, steamed with no added fat	100	g	0.111
Oyster, aquacultured, raw	100	g	0.445
DISCRETIONARY FOODS	SERVING		NRF-ai score
Soft drink, cola flavour	375	g	-0.135
Biscuit, sweet, Anzac style, homemade from basic ingredients	35	g	-0.020
Biscuit, sweet, biscuit base, mint filling, chocolate-coated	35	g	-0.006
Doughnut, jam filled, sugar coated	40	g	0.000
Muffin, cake-style, berry, commercial	40	g	0.004
Ice cream, vanilla flavour, regular fat	75	g	0.024
Ice confection, stick, milk-based, various flavours	75	g	0.015
Ice confection, stick, water-based, various flavours	75	g	-0.035
Ice confection, stick or tub, fruit juice or fruit flavoured	75	g	-0.034
Corn chips, plain, toasted, salted	30	g	0.032
Potato crisps or chips, plain, salted	30	g	0.023
Chocolate, milk	25	g	0.007
Chocolate, white	25	g	-0.003
Jam, berry	30	g	-0.052
Lolly, jelly varieties	40	g	-0.052
Lolly, mint flavoured, sugar sweetened	40	g	-0.084
Liquorice, allsorts	40	g	-0.048

Nutrient composition data sourced from Australian Food Composition Database: https://www.foodstandards.gov.au/science/monitoringnutrients/afcd/pages/default.aspx
